# Communicating pain: emerging axonal signaling in peripheral neuropathic pain

**DOI:** 10.3389/fnana.2024.1398400

**Published:** 2024-07-09

**Authors:** Livia Testa, Sofia Dotta, Alessandro Vercelli, Letizia Marvaldi

**Affiliations:** ^1^Neuroscience Institute Cavalieri Ottolenghi, Orbassano (Torino), Torino, Italy; ^2^Department of Neuroscience “Rita Levi-Montalcini”, Torino, Italy

**Keywords:** neuropathic pain, peripheral nerve injury, neurogenetics, axonal signaling, dorsal root ganglia, axonal regeneration, nerve regeneration

## Abstract

Peripheral nerve damage often leads to the onset of neuropathic pain (NeuP). This condition afflicts millions of people, significantly burdening healthcare systems and putting strain on families’ financial well-being. Here, we will focus on the role of peripheral sensory neurons, specifically the Dorsal Root Ganglia neurons (DRG neurons) in the development of NeuP. After axotomy, DRG neurons activate regenerative signals of axons-soma communication to promote a gene program that activates an axonal branching and elongation processes. The results of a neuronal morphological cytoskeleton change are not always associated with functional recovery. Moreover, any axonal miss-targeting may contribute to NeuP development. In this review, we will explore the epidemiology of NeuP and its molecular causes at the level of the peripheral nervous system and the target organs, with major focus on the neuronal cross-talk between intrinsic and extrinsic factors. Specifically, we will describe how failures in the neuronal regenerative program can exacerbate NeuP.

## 1 Introduction to neuropathic pain

### 1.1 What is Neuropathic pain?

With the term “pain” we refer to an “unpleasant sensory and emotional experience that is associated with or resemble actual or potential tissue damage,” as defined by the International Association for the Study of Pain (IASP; Raja et al., 2020). We consider pain a debilitating condition, but in reality this is an evolutionary conserved protective response to harmful stimuli, such as excessive cold/heat, chemical irritants and dangerous mechanical forces (Testa et al., 2021). Indeed, patients with congenital insensitivity to pain suffer from multiple lesions, untreated bone fractures and severe complications (Phatarakijnirund et al., 2016; Wang et al., 2016; Hartono et al., 2020). Nonetheless, excessive pain is detrimental and pharmacological treatments are necessary to abate it.

We distinguish two phases of pain: acute and chronic. Acute pain arises from chemical exposure (acetone, capsaicin), temperature (heat and cold) and mechanical stimuli (Fernandez Rojas et al., 2023). Acute pain is the first response to damaged tissue, followed by inflammation that triggers swelling of the area and promotes tissue repair (Rabiller et al., 2021; Parisien et al., 2022). However, if the pain persists for more than 3 months, it is defined as chronic and it becomes a pathological condition in itself (Treede et al., 2019). A temporal parameter is used to differentiate between acute and chronic pain due to the lack of consistent biomarkers that could be applied in the clinical setting.

Pain is also classified according to its origin as nociceptive (when tissues are injured), neuropathic (if nerves are damaged) or nociplastic (when the nervous system is sensitized, while no damages are observed on tissues and/or peripheral nerves; Fitzcharles et al., 2021). In the clinical practice, it may be difficult to separate the types of pain and most of the conditions may present a mixed phenotype such as neuropathic and nociplastic (Caraceni and Shkodra, 2019).

Neuropathic pain (NeuP) can arise because of lesions or diseases (genetic or acquired) affecting the somatosensory nervous system (SNS). The SNS is called spinothalamic tract ascending pathways formed by the synapses of three order of neurons: primary neurons (housed in dorsal root ganglia), secondary neurons (located in the spinal cord) and tertiary neurons (present in the thalamus; Viswanath et al., 2020; LRM et al., 2021). This system is responsible for the perception of crude touch, pain, temperature, as it integrates information from external stimuli and conveys them from the periphery to the cerebral cortex. Any damage of this pathway disrupts the signal transmission and can results in pain (Colloca et al., 2017).

### 1.2 Pathologies associated with peripheral NeuP

While acute trauma is a common trigger, NeuP can develop from non-traumatic conditions that affect the nervous system. These conditions may be: (a) genetic mutations or polymorphisms, (b) acquired afflictions, like infections or injuries, or (c) medical treatments or drugs.

In humans, mutations of certain genes, such as PMP22, GJB1, MPZ and GDAP1, cause Charcot–Marie–Tooth disease, a group of inherited disorders characterized by nerve damage with painful motor and sensory neuropathy (Liu et al., 2020). People suffering from erythromelalgia and paroxysmal extreme pain disorder (Ahn et al., 2013; Goodwin and McMahon, 2021), generally called idiopathic painful small fiber neuropathies, present gain-of-function mutations in sodium voltage-gated channel encoding NaV1.7, NaV1.8, and NaV1.9. Other mutation in TRPA1, TRPV1, α-galactosidase and KIF5A (Biegstraaten et al., 2012; Boukalova et al., 2014; Rinaldi et al., 2015) are responsible for sensory neurons hyperexcitability that clinically manifests as sudden bouts of pain propagating inward from the extremities. Mutations of SPTLC1, a serine palmitoyltransferase, cause a form of hereditary sensory neuropathy with early sensory loss and later “lightning” or “shooting” pains (Lorenzoni et al., 2023). More gene variations have been associated to the development of painful syndromes, as reported in the DOLORisk study^1^ (Pascal et al., 2019) and the Human Pain Genetics Database (HPGDB; humanpaingeneticsdb.ca; Meloto et al., 2018).

A plethora of acquired afflictions can damage nerves and provoke NeuP. This is the case of spinal cord injury (Shiao and Lee-Kubli, 2018), diabetes (Feldman et al., 2019), herpes zoster infection (Kinchington and Goins, 2011), HIV infection (Laast et al., 2011), Lyme disease (Karri and Bruel, 2021) and also COVID infection (Fernández-De-las-peñas et al., 2022). Cancer may induce NeuP by compressing the surrounding nerves while growing or by inducing fibrosis, both of which cause pain fibers hypersensitivity (Oh and Yoon, 2018). Moreover, the pro-inflammatory cytokines released by the immune cells recruited in the tumor microenvironment may increase pain perception and hyperalgesia (Bennett et al., 2012; Caraceni and Shkodra, 2019).

People suffering from painful conditions often turn to surgical or pharmacological treatments, but they may not always find relief. Surgical operations cause additional nerve damage, which can evolve in persistent Surgically-Induced Neuropathic Pain (SNPP; Borsook et al., 2013). Drugs used to treat pain, such as psychotropic and anticonvulsants (e.g., gabapentin; Jones et al., 2019), can trigger Drug Induced Peripheral Neuropathy (DIPN). Chemotherapy-induced peripheral neuropathy (CIPN) can cause irreversible nerve damage with pain that cannot be relieved even after the end of the treatment (Zhang et al., 2016; Bjornard et al., 2018; Eldridge et al., 2021). In particular, CIPN patients present altered activity and expression of voltage-gated ion channels (i.e., neurotransmission) and loss of intraepidermal nerve fibers and Meissner’s corpuscles in the skin (Boyette-Davis et al., 2015). Understanding the molecular basis of neuropathic pain to develop targeted analgesic could be incredibly beneficial for all these patients.

### 1.3 Epidemiology of NeuP

**Figure 1:**
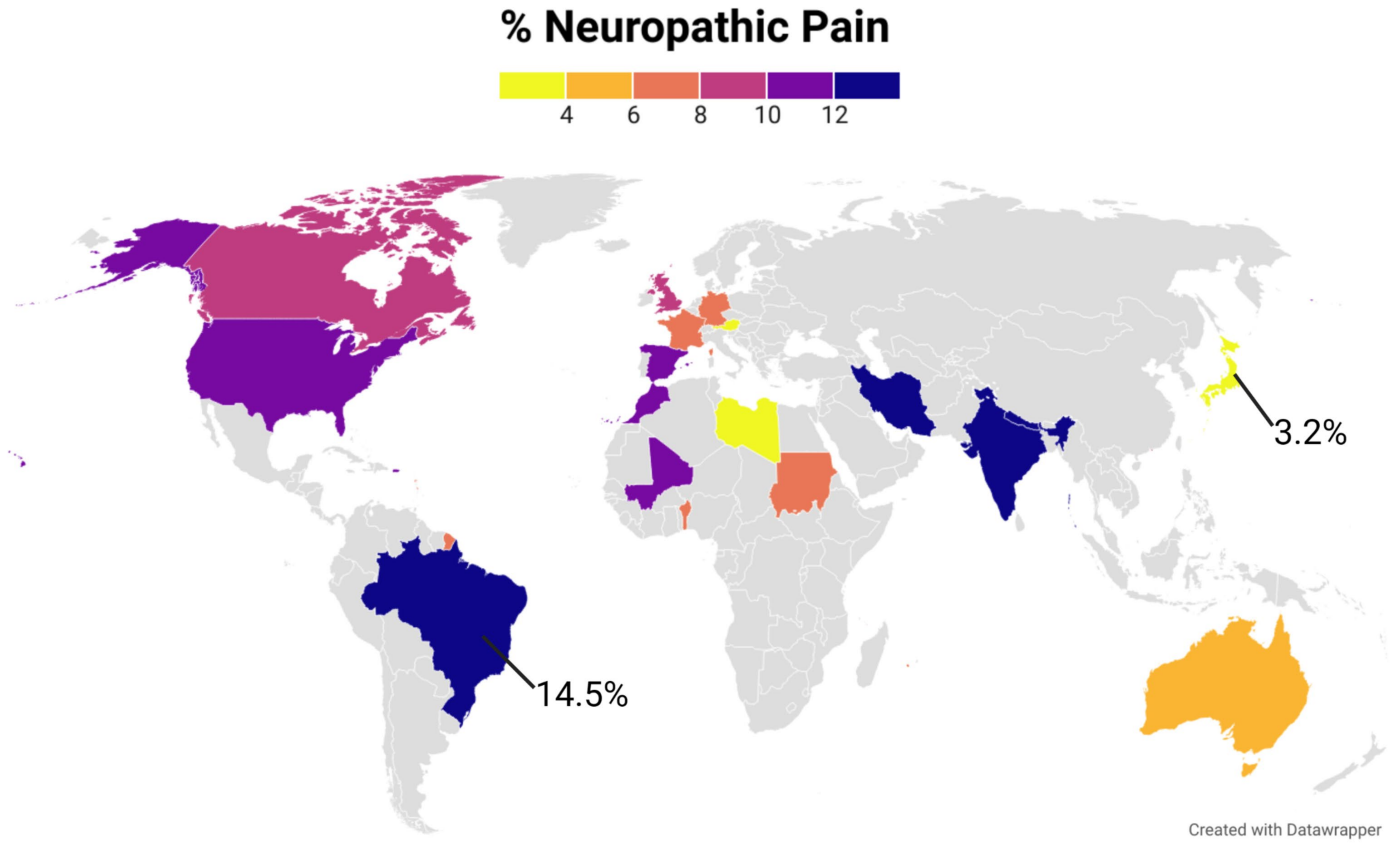
The world map represents the prevalence of NeuP in different countries in the general population. A color gradient was used for those countries with published data. Gray color was used for countries were no studies were found. The figure was created using Datawrapper, and is also available online at the following link https://datawrapper.dwcdn.net/kgAz0/1/. Additional information on the studies used to compile this graph is available in the Supplementary Table 1.

It is estimated that between 6.9% and 10% of the world general population suffers from chronic NeuP (Van Hecke et al., 2014). The prevalence reported in population studies varies between 3.2% and 14.5%, likely due to differences in evaluation methods, language barriers, sample recruitment processes, and patient self-reported information employed in the data collection (Figure 1).

Clinical diagnosis (e.g., documented neurological lesion) is rarely used in the census studies due to the difficulties in the patients’ recruitment process. The majority of the epidemiologic investigations employ one of three screening questionnaires for NeuP assessment: PainDETECT, LANSS (Leeds Assessment of Neuropathic Symptoms and Signs), or DN4 (Douleur neuropathique 4). Even though their results do not completely overlap (VanDenKerkhof et al., 2016; Attal et al., 2018), these questionnaires are a useful tool to identify the classical symptoms of NeuP, specifically allodynia (i.e., pain by a stimulus that should not be causing discomfort), hypersensitivity, shooting pain, numbness, burning and tingling sensations (Truini et al., 2013).

NeuP symptoms greatly affect the quality of life of the people and increase the individual healthcare cost. Looking at five European countries (Italy, Spain, France, the UK and Germany), the average annual spending healthcare-related per patient ranged from €1,939 to €3,131, when adjusted to 2012 prices (Liedgens et al., 2016). Additionally, psychological factors (such as stress, anxiety, and depression) can worsen and, at the same time, be worsened by neuropathic pain (Breivik et al., 2013; Meng et al., 2020; Roughan et al., 2021). This psychological spiral is usually attenuated when the patients have a support system around them to help dealing with pain (Cohen et al., 2021).

Consumption of western-style high-fat diet, excessive alcohol and sedentariness are on the rise worldwide, and unfortunately they are also correlated with an increased risk of developing NeuP (Brandão et al., 2020; Dudek et al., 2020; Smith et al., 2020; Tanaka et al., 2023). Therefore, there is an urgent need for proper unbiased pain biomarkers to be employed in the clinics to diagnosis and then alleviate pain in the sufferers.

### 1.4 Sex hormones and the effect of aging on NeuP

It is widely known that pain perception varies according to age, sex, and ethnic group (Mills et al., 2019; Chang et al., 2022). Females more than males suffer from NeuP, a phenomenon observed in both rodents and humans (Szabo-Pardi et al., 2021; Elliott et al., 2024). Sex hormones are known to influence pain perception, as both estrogen and testosterone receptors are expressed in sensory neurons. In particular in peripheral nociceptors, 17-β-estradiol increase sensitivity to mechanical and thermal pain (Patrone et al., 1999; Deng et al., 2017), while testosterone, binding to TRPM8, dampens pain perception (Barbosa Neto et al., 2019). The molecular bases of sex-dimorphism in NeuP are still unclear, but it has been speculated that sex steroids might influence specific protective or detrimental gene expression for pain perception (Stephens et al., 2019) and axonal regeneration (Ward et al., 2021).

Population studies indicate that NeuP is prevalent in the elders. This does not mean that young people are exempt from nerve damage. In both humans and animals, the nerve damage occurring at an early age will trigger NeuP only in late childhood and adolescence (Walco et al., 2010). In rats, specifically, nerve injuries before P28 will develop into NeuP only after 3 weeks, a time that corresponds to the animal’s adolescence (Fitzgerald and McKelvey, 2016). This phenomenon occurs because before P28 the neuroimmune response is skewed toward anti-inflammation, which suppresses nociceptors excitability and prevents NeuP. As the rodent grows, the neuroimmune signature shifts toward pro-inflammation, which uncovers the latent pain response to early trauma (McKelvey et al., 2015).

In general, with age there are increased number of abnormal or degenerating neuronal fibers, slower conduction speed, altered endogenous inhibition and decreased function of neurotransmitters, all of which favor NeuP development (Giovannini et al., 2021). Nociceptor gene expression also changes with age. Aged murine models (18–24 months) have increased pain sensitization (Tac1 and Calca) and stress (Atf3) markers in DRGs, and also elevated levels of neurotrophic factor Bdnf (Vincent et al., 2020).

The described physiological variability renders pain detection and analgesic development incredibly challenging. It will be difficult to develop an all-encompassing wonder drug to resolve NeuP in all the conditions for all type of patients. Pharmacological studies, especially, will have to be even more attentive in subject clustering to properly identify drug candidates.

## 2 DRG neurons in NeuP

### 2.1 DRG structure

Animals perceive pain, defined as intense above threshold thermal, mechanical or chemical stimuli, via a subpopulation of peripheral nerve fibers called nociceptors (Basbaum et al., 2009), that are to the primary order neurons mentioned previously. These nociceptors have their soma situated in the dorsal root ganglia (DRG), bilateral structures that reside inside the intervertebral foramina. Therefore, DRGs are functional centers for sensory transduction and modulation, but also for pain transmission and maintenance of pain states (Berger et al., 2021). The neurons residing in the DRG structures are a population heterogeneous in size and function. In the same DRG it is possible to recognize nociceptors’, mechanoceptors’ and propioceptors’ cell bodies (Belmonte and Viana, 2008), which present diverse gene expression profiles. Through single cell-sequencing, several researchers could even obtain the transcriptome signature of the different DRG nociceptors, a throve of information available online in several databases (Table 1).

**Table 1:** Online databases of DRG transcriptome.

**Database**	**Laboratory**	**Tissue and cell origin**	**Details of the study**
**SeqSeek**SeqSeek (nih.gov)	N. Ryba, NIHA.J. Levine, NIHNguyen et al. (2021), Russ et al. (2021)	Human DRGMouse spinal cord	Map of human DRG, according to functionMurine spinal cord cell atlas
**Sex difference in pain**Resources — Denk Laboratory (franziskadenk.com)	F. Denk, King’s College LondonLopes et al. (2017)	Mouse DRG	Male and Female mouse nociceptorsComparison between naive and injury states
**NIPPY - Neuro-Immune interactions in the Periphery** http://rna-seq-browser.herokuapp.com/	F. Denk, King’s College LondonLiang et al. (2020)	Mouse sciatic nervesMouse DRG	Male and Female mouse nociceptors and sciatic nerveComparison between naive and injury states
**Sensoryomics (DRG TXome Database)** https://sensoryomics.shinyapps.io/RNA-Data/	T.J. Price, University of Texas (Dallas)Tavares-Ferreira et al. (2022)	Human nociceptors	DRG transcriptomic Neuropathic pain
**Nociceptra**Streamlit (nociceptra.streamlit.app)	M. Kress, Medical University InnsbruckT.J. Price, University of Texas (Dallas)Zeidler et al. (2023)	Human iPSC-derived sensory neurons	Expression Signatures
**XSpecies DRG Atlas**XSpecies DRG Atlas (gene.com)	L. Riol-Blanco, GenentechJ.S. Kaminker, GenentechD.H. Hackos, GenentechJung et al. (2023)	Mouse DRGGuinea pig DRGMonkey DRGHuman DRG	Cross-species transcriptome atlas of dorsal root ganglia (naive)
**Harmonized DRG and TG reference atlas** https://painseq.shinyapps.io/harmonized_drg_tg_atlas/	W. Renthal, Brigham and Women’s Hospital and Harvard Medical SchoolR.W. Gereau IV, Washington University School of MedicineT.J. Price, University of Texas (Dallas) Bhuiyan et al. (2023)	Human DRG and TGFive other species DRG and TG	Cross-species transcriptome atlas of DRG and TG (naive)Neuronal and non-neuronal cells

Nociceptors have their soma enveloped by satellite glial cells (Avraham et al., 2022; Mapps et al., 2022), that are multipotent glial precursors implicated in pain transmission. The axons of these sensory neurons are in close association with myelinating or non-myelinating Schwann cells (Harty and Monk, 2017). Nociceptors with myelinated axonal projections are termed Aδ-fibers (1-5 μm diameter), while those lacking myelin wrapping are C-fibers (0.2–1.5 μm diameter). In the distal peripheral nerve, C-fibers are closely associated with non-myelinating Schwann cells, forming Remak bundles, that are structures crucial for neuronal repair after peripheral nerve injury (Harty and Monk, 2017). The two types of fibers serve different functions: Aδ-nociceptors elicit fast, sharp pain (“first pain”) after mechanical and chemical stimuli; C-nociceptors transmit slow, aching dull pain (“second/slow pain”) following an ample range of stressors (i.e., polymodal function; Basbaum et al., 2009).

**Figure 2:**
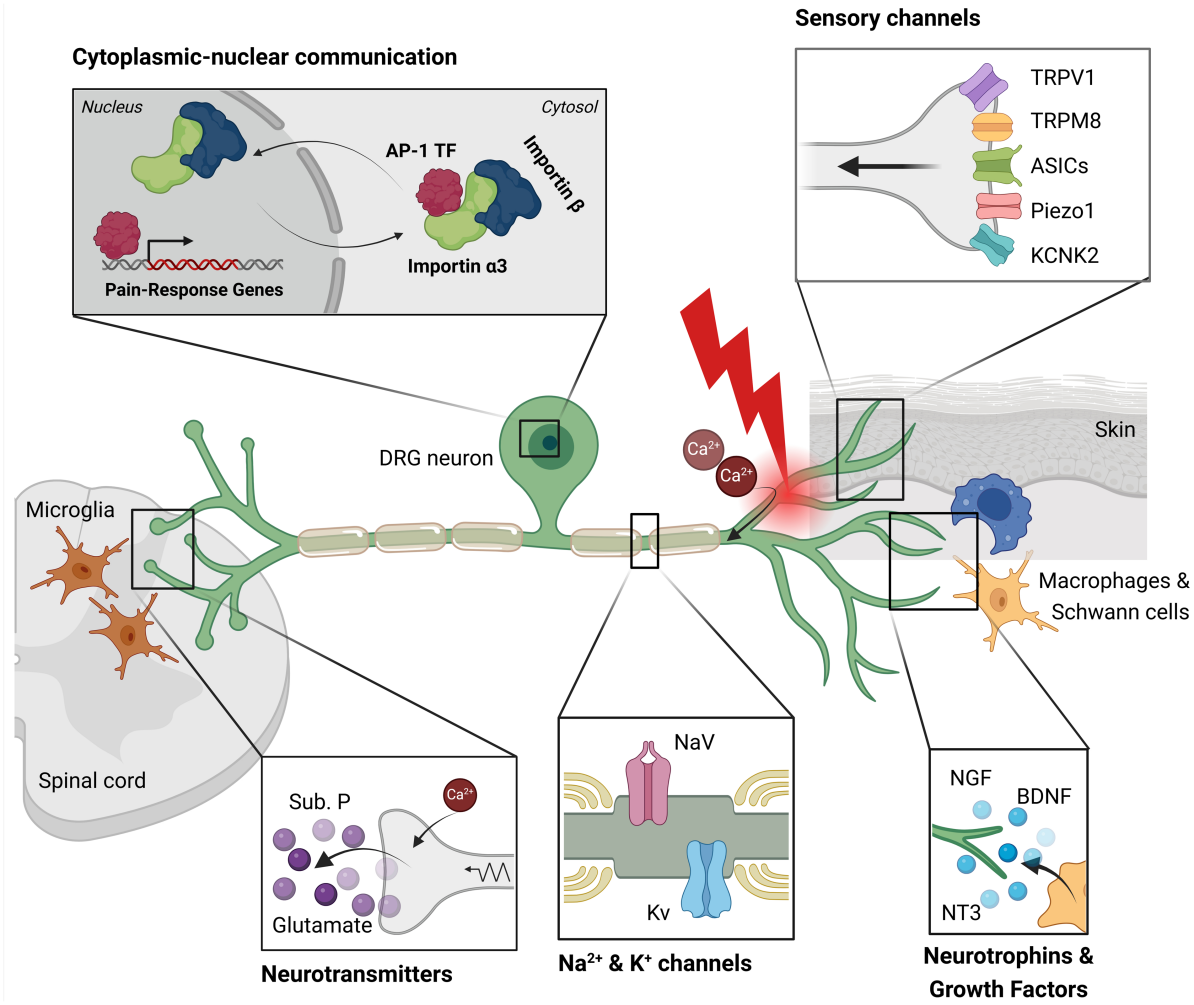
This illustration depicts key components involved in pain signaling within a dorsal root ganglion (DRG) neuron. Sensory channels located in the peripheral axon detect mechanical, thermal (heat/cold), and chemical stimuli. Resident macrophages and Schwann cells near the neuron release neurotrophins, cytokines, and growth factors, which support cell survival. Following axotomy, calcium ions enter the neuron, initiating an injury signal. This signal activates sodium and potassium channels, which transmit the signal towards the nucleus. Transcription factors, such as AP-1, bind importin α3 and are transported into the nucleus, where they induce the expression of genes associated with pain and axonal regeneration. Additionally, the injury signal is conveyed to the central nervous system via saltatory conduction. At the synapse with the second-order neuron, the calcium influx in the sensory neuron triggers the release of substance P and glutamate. Activated pro-inflammatory microglia in the surrounding region amplify mechanical hypersensitivity and pain. This image was created with BioRender.

DRG neurons possess a peculiar morphology: *in vivo* they are bipolar in shape during the embryonic stage, while upon maturation they become pseudo-unipolar (Nascimento et al., 2018), with a single axon—the stem axon—that bifurcates (Figure 2). The peripheral branch innervates skin, muscle and viscera and acts as the afferent portion of the system, while the central branch reaches the dorsal horn of spinal cord (laminae I and II) where it synapses with second-order neurons (Basbaum et al., 2009; Nascimento et al., 2018). These spinal neurons project via the spinothalamic tract to upper brain structures (like the cerebral cortex) to transmit noxious stimuli and information about intensity and location. Some of the secondary order neurons project to the cingulate and insular cortex via the connections in the parabrachial nucleus and the amygdala, contributing to the pain experience (Yam et al., 2018).

The DRG structure contains other non-neuronal cells, such as macrophages and T-lymphocytes and a small number of B-lymphocytes (Laast et al., 2011; Makker et al., 2017; Zhou et al., 2022; Feng et al., 2023). Endothelial and smooth muscle cells are also present, as fenestrated capillaries directly irrorate the DRGs to release oxygen and blood borne molecules that interact with the neuronal cells (Jimenez-Andrade et al., 2008). These surrounding cells and their released factors directly influence the functions of the sensory neurons.

### 2.2 Nociceptor signaling after peripheral nerve injury

In general, NeuP arises when nerves are damaged. Rupturing the nociceptor plasma membrane triggers signaling cascades that alter the expression and function of ion channels. This causes a change in the electrical signal transmission, which the nervous system processes and perceives as pain.

When the nociceptor peripheral branch undergoes axotomy (Figure 2), the distal axon is separated from the cell body and is subjected to Wallerian degeneration, an active process that disrupt the axolemma. At the same time, the proximal axon is exposed to inflammatory cytokines and trophic factors from the surrounding cells (e.g., Schwann cells, macrophages; Campbell and Meyer, 2006; Rotshenker, 2011), which activate signaling cascades. Axotomy does not activate ion channels like TRPV1 (heat), TRPM8 (cold), ASICs (acidic milieu), TRPA1 (chemical irritant), KCNK2/TREK-1 (mechanical stimuli) and Piezo1 (mechanical stimuli; Wang and Woolf, 2005; Patapoutian et al., 2009; Coste et al., 2010; Wemmie et al., 2013; Djillani et al., 2019).

The plasma membrane rupture leads to ionic influx, elevated intracellular calcium levels, and cytoskeleton disruption through calpain activation (George et al., 1995; Zang et al., 2015). Apart from calpain activity, the axotomy causes actomyosin contraction, which makes the sensory neurons shrink. This is necessary to eliminate water via aquaporin channels and to prevent excessive swelling that may lead to cell death (Aydın et al., 2023). The calcium wave moves toward the soma to trigger epigenetic changes and regeneration-associated genes (RAGs) expression. Slow motor-based retrograde complexes deliver injury signaling (such as ERK) to the nucleus (Puttagunta et al., 2014). Axon injury activate other molecular pathways such as cAMP/PKA, PTEN/mTOR, gp130/Jak and DLK/JNK (Zigmond, 2012; Li et al., 2015; Valakh et al., 2015; Chen et al., 2016; Alber et al., 2023), all of which target transcription factors (such as ATF3, CREB, STAT3, and c-Jun) to promote regeneration (Bareyre et al., 2011; Moore and Goldberg, 2011). Gene inactivation mediated by DNA methylation as well as gene downregulation by non-coding RNA transcripts (miRNAs, siRNAs, lncRNAs) are involved in the control of the axon regeneration program (Oh et al., 2018; Han et al., 2022). Interestingly, these regenerative programs are sexually dimorphic in the early phases (Chernov and Shubayev, 2022). Several alterations in gene expression post-nerve injury have been reported in a number of studies, some of which are reported in Table 1.

As mentioned, the calcium influx is necessary to induce the initial neuronal survival program. However, if the calcium influx persists, it lowers the threshold for action potentials, making the DRG neurons hyperexcitable, which favors NeuP development (Chung and Chung, 2002). Even sodium and potassium ionic currents, propagating along the axons through specific channels, are involved in pain signaling. In sensory neurons, the main sodium channels are NaV1.7, NaV1.8 and NaV1.9, while the potassium ones are Kv1.2, TRAAK and TREK-1: improper activities of these channels can lead to either hyperalgesia or analgesia (Tsantoulas and McMahon, 2014; Goodwin and McMahon, 2021). Ionic currents travel toward the central axonal branch that forms a synapse with the second-order neurons in the dorsal horn of the spinal cord (Todd, 2010). Here, the calcium influx triggers the release of neurotransmitters and neuropeptides (such as glutamate, substance P and CGRP), that will be captured by the spinal cord neurons and transmitted to the CNS (Gross and Üçeyler, 2020).

When nociceptor activation is persistent, neural circuits undergo rearrangements. Changes have been observed in genes and proteins expression which affect neuronal excitability and transmission (i.e., functional plasticity), in the spines morphology (i.e., structural remodeling) and in the neural connectivity (Tracey et al., 2019; Fiore et al., 2023). The result of these alterations is sensitization to pain, either at the peripheral or central levels, which can be aggravated by the pro-inflammatory products released by surrounding cells (Woller et al., 2017; Rosenbaum et al., 2022).

## 3 Neuronal factors contributing to NeuP post injury

### 3.1 Retrograde transport and importins

As mentioned, damaged sensory axons activate two response phases: an early one, mediated by ion influxes (mainly calcium), and a late one, characterized by slower signals conveyed through molecular motors. These molecular motors travel on microtubules and move vesicles, organelles, proteins, and RNA granules containing snRNP along the axons (Rishal and Fainzilber, 2014; Saito and Cavalli, 2016; Smith et al., 2020). There are two type of motor proteins: the plus-end directed kinesins, and the minus-end directed dynein. In neurons, dynein exclusively moves cargo from pre-synapses back to the soma (Terenzio et al., 2020), a process called “retrograde transport.” Retrograde transport is essential for regulating cell homeostasis, neurotrophic factor signaling, autophagy–lysosomal degradation, nerve injury response and pain signaling (Rishal et al., 2012; Rishal and Fainzilber, 2014; Prior et al., 2017; Mao et al., 2019; Marvaldi et al., 2020). Indeed, reduced expression of the dynein heavy chain 1 (Dync1h1) in sensory and motor neurons causes accelerated axonal outgrowth and delayed recovery after injury (Di Pizio et al., 2023).

Protein kinase signaling pathways and post-translational microtubule modifications regulate the efficiency of retrograde transport (Barlan and Gelfand, 2017; Brady and Morfini, 2017). To properly function, retrograde axonal transport requires the interaction between the dynein motor and its cargo, which is usually mediated by adaptor proteins. Adaptor/scaffold proteins dictate the specificity of the cargoes to be shuttled. Any deregulation caused by modifications of key adaptors and scaffolds could result in neuropathic pain. Indeed, some forms of hereditary Charcot–Marie–Tooth have mutations that compromise retrograde transport (Markworth et al., 2021).

**Figure 3:**
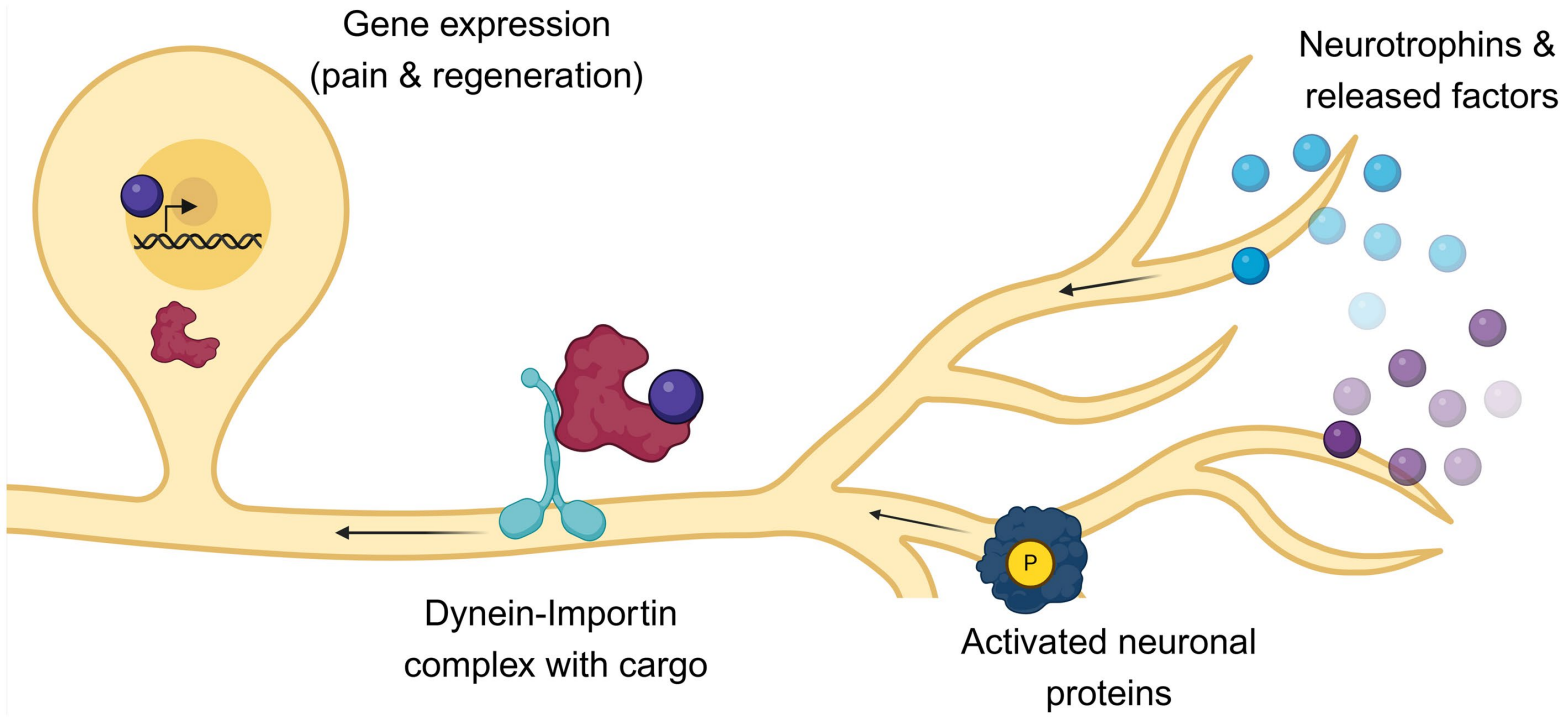
Following axotomy, the fate of nociceptors is influenced by both extrinsic and intrinsic factors. After axonal injury, pro-survival factors such as neurotrophins are released by surrounding cells. Concurrently, specific molecular cascades are activated within the damaged neurons. These signals must be transported from the periphery to the nucleus to initiate pain and axonal regeneration signaling. This retrograde transport is facilitated by the motor protein dynein and its adaptor importin, which are localized along the axons and shuttle cargoes to the nucleus of nociceptors. Any impairment in this retrograde transport can affect both pain perception and axonal recovery. This image was created with BioRender.

Importins are a family of adaptor proteins involved in retrograde transport (Figures 2, 3). These proteins, classified as karyopherins, are divided into ɑ and β subunits. To be functional, importins form heterodimers, where β interacts directly with dynein while ɑ binds the nuclear localization signals (NLS) of cargo proteins (Panayotis et al., 2015). Moreover, importin β mediates the docking of the importin/substrate assembly to the nuclear pore complex (NPC) through binding to nucleoporin FxFG repeats (Lott and Cingolani, 2011).

Mouse and human have, respectively, six and seven isoforms of importin ɑ, with specific tissue expression profiles and cargo-binding selectivity. For example, importin ɑ5 directly binds and regulates nuclear import of MeCP2, which affects anxiety levels (Panayotis et al., 2018). Importins ɑ1 and ɑ5 were also found to bind viral proteins to aid viral replication of herpes simplex virus and Newcastle disease virus, respectively (Döhner et al., 2018; Duan et al., 2018). Interestingly, mutant importin ɑ4 can cause Infantile-Onset Hereditary Spastic Paraplegia, though the molecular mechanism is unclear (Schob et al., 2021). Importin ɑ3 was recently found to be relevant for persistence of chronic NeuP (Marvaldi et al., 2020).

### 3.2 Cargos in response to nerve injury

In both naive and injured sciatic nerve, importin α-s are in axons constitutively associated with dynein, while importin β1 protein assumes axonal localization only after an injury occurred (Hanz et al., 2003; Alber et al., 2023). Moreover, only upon damaged importin β undergoes local axonal translation and forms α/β functional heterodimers to accelerate the retrograde transport of cargo (Perlson et al., 2005).

What are the cargoes that are retrogradely transported after axonal injury? Transcription factors (TF), such as ATFs and STATs, have been found to used importin-based nucleocytoplasmic transport (Lindwall and Kanje, 2005; Michaelevski et al., 2010). Members of AP-1 family of TF, which have roles in neuronal activation and axonal regeneration, also bind importins (Raivich et al., 2004). In particular c-FOS, a member of the AP-1 group, binds importin α3-β complex, which results in its nuclear import and the expression of downstream genes that regulate pain (Manassero et al., 2012; Marvaldi et al., 2020). Mice injected with AAV9 vector [that specifically targets sensory neurons (Chan et al., 2017)], carrying importin α3 shRNA had reduced pain perception in the acute and chronic pain response. Coherently, blocking the nuclear import of AP-1 factors was sufficient to reduce pain. This effect was even reproduced pharmacologically with the use of two non-analgesic FDA-approved drugs (sulmazole and sulfamethizole), identified via cMAP screening analysis (https://www.broadinstitute.org/connectivity-map-cmap Connectivity Map (CMAP) | Broad Institute). Further analysis showed that indeed these two drugs reduced pain by blocking c-Fos nuclear import (Marvaldi et al., 2020).

STAT3 is another TF that not only is locally translated in the axon and activated upon injury, but also retrogradely transported by dynein-importin α5. This modulates survival of sensory neurons *in vivo* by acting as an anti-apoptotic factor (Ben-Yaakov et al., 2012). Experimental evidence suggests that even members of the Myc/Max, PPAR and Smad families may undergo the same dynein-mediated transport in rodents after sciatic nerve injury (Ben-Yaakov et al., 2012), though additional biochemical assay will be required to obtain a full picture of the phenomenon.

Signaling endosomes are another kind of cargoes retrogradely transported after injury via dynein motors. The maturation and movement of these endosomes are regulated by Rab5/Rab7 and Erk1/2 (Deinhardt et al., 2006; Ito and Enomoto, 2016). Specifically, Rab5 is found associated with stationary organelles, while Rab7 is present in the moving endosomes. In sensory neurons, the tethering of the signaling endosomes to dynein motor protein is mediated by retrolinkin (a membrane endosomal protein) that directly binds BPAG1n4, which in turn is associated with dynactin/dynein (Liu et al., 2003, 2007).

As they originate from the plasma membrane, the signaling endosomes are responsible for endocytosis of ligand and their receptors, such as P2X3, NaV1.7 and Trk receptors (Chen et al., 2012; Higerd-Rusli et al., 2023). Interestingly, P2X3, a ATP-receptor highly expressed in DRG nociceptors, has been associated with neuropathic pain and its pharmacological downregulation has showed analgesic effects in rat (Dan et al., 2021). TrkA-NGF complexes are endocytosed and retrogradely transported together with CREB TF (that was locally translated) and other signaling molecules like MEK, ERK, PLCγ and PI3K (Cosker et al., 2008; Marlin and Li, 2015; Crerar et al., 2019). By doing so, the signaling endosomes effectively become platforms for the propagation of molecular cascades that got activated a the nerve terminal. The CREB TF contained in the vesicles, once delivered in the proximity of the nucleus, activates genes for neuronal survival (Cox et al., 2008; Melemedjian et al., 2014). Alteration of this signaling pathway was observed in Charcot–Marie–Tooth mice models carrying Gars mutations and, as expected, these mice also display sensory defects (Sleigh et al., 2017).

In DRG neurons, other neurotrophins-receptor complexes, such as BDNF-TrkB (Vermehren-Schmaedick et al., 2022) undergo similar retrograde transport prompting the expression of anti-apoptotic/pro-survival genes that prevent nerve degeneration.

### 3.3 Axonal regeneration post-injury is altered by NeuP

The injury signals delivered through retrograde transport induce alterations of the cytoskeletal architecture and of the gene expression profile (Renthal et al., 2020). These rearrangements require epigenetic changes dependent on the activity of MeCP2, DNMTs, and on the export of HDAC5 (Cho et al., 2013; Penas and Navarro, 2018).

The nuclear import of transcription factors [e.g., Jun, ELK1, STAT3, SMAD (Doron-Mandel et al., 2015)] promotes the expression of several genes associated with regeneration, such as Atf3, Sprr1a, Gap43, Sox11, Gadd45a, Smad1 and NPY (Jang et al., 2021). Gap43, a well-known protein involved in axonal growth, also increases in the axons following the local translation of mTOR (Terenzio et al., 2018). Axonal regeneration is promoted by reduced levels of molecules, such as Spry2, Sarm1, Gas5 and DRAK2 kinase, that regulate the activity of growth factor receptors and inflammatory pathways (Marvaldi et al., 2014, 2015; Thongrong et al., 2016; Han et al., 2022; Park et al., 2023). In the growth cone, the axonal elongation is at the same time stabilized by p110δ PI 3-kinase and destabilized by RhoA/ROCK (Eickholt et al., 2007), while the branching relies MAP7 and Sema3A signaling (Terenzio et al., 2018; Hu et al., 2021). The directionality of the axonal growth is controlled by gradients of cytokines and growth factors (released by the surrounding cells; Turney et al., 2016; Grasman and Kaplan, 2017), that regulate Slit/Robo and Netrin/DCC signaling pathways (Yi et al., 2006; Webber et al., 2011).

Axonal regeneration requires all these steps and more, however actual functional recovery is slow, often incomplete and accompanied by NeuP. Moreover, some transcription factors that promote axon growth also contribute to NeuP development. Among these TFs are listed the previously mentioned Jun/Fos, but also the upregulated OCT1 and the downregulated EBF1 and NRF2 (Yuan et al., 2019; Vasavda et al., 2022; Liang et al., 2024). Perturbation of guidance molecules gradients and altered axonal sprouting, which lead to impaired pathfinding and tissue mistargeting, can cause NeuP (Xie et al., 2017; Gangadharan et al., 2022). Painful neuromas are one of the most common clinical manifestation of erroneous target innervation (Shamoun et al., 2022).

### 3.4 Altered gene expression by non-coding RNA after injury

The retrogradely-transported transcription factors are not the only elements that perturb gene expressions in sensory neurons after injury. Altered levels of non-coding RNA (ncRNAs), mainly miRNA and lncRNA, have been associated with neuropathic pain. Some ncRNAs have been even proposed as NeuP biomarkers, but significative differences were observed between in vivo and in vitro experiments (Hu et al., 2021), invalidating their widespread use.

Functionally, ncRNAs expressed by sensory neurons act at the post-transcriptional level to modulate the expression of proteins involved in the injury/regenerative response. For example, miR-21 and miR-222, which are found elevated in rat DRG post sciatic nerve injury, downregulate TIMP3, a pro-apoptotic protein, and promote neuronal viability (Strickland et al., 2011; Zhou et al., 2015). Few miRNAs have been identified to affect DRG neurons, by either favoring or impairing the axonal elongation. Among these figure miR-132, that by targeting RASA1 promote axonal extension (Hancock et al., 2014), and miR-138, which is downregulated in injured DRG neurons as it suppress axonal growth by targeting SIRT1 (Liu and Wang, 2013). Even lncRNAs found in DRG post nerve injury, such as lncRNA BC089918, were found to affect neuronal growth (Yu et al., 2013).

Few ncRNAs have been found deregulated in murine models of NeuP. In particular, in rat DRG the expression of several potassium channels was compromised by the upregulation of miR-18a, miR-19a, miR-19b, and miR-92a (Sakai et al., 2017). Both miR-30b and miR-182, highly expressed in NeuP developed post nerve injury, could reduce the amount of NaV1.7 and alleviate NeuP (Shao et al., 2016; Cai et al., 2018). Ion channels are not the only targets of ncRNA in NeuP conditions. In rat with constricted nerves, miR-206 favors analgesia by physiologically reducing the levels of BDNF (Sun et al., 2017). On the other hand, lncRNA LINC01119, upregulated in NeuP conditions, binds BDNF mRNA and stabilizes it, promoting hypersensitivity (Zhang et al., 2021).

Human pathologies with NeuP symptoms display altered expression of ncRNAs. For examples, in the patients’ blood miR-34a and miR-101 were downregulated, while miR-199a-3p and miR-455-3p were upregulated (Shenoda et al., 2016; Li et al., 2017; Asahchop et al., 2018; Liu et al., 2019). Interestingly, reduced levels of miR-101 correspond to an increase of importin β protein (which is the miRNA direct target) and to the activation NF-κB signaling, which contributes to NeuP development (Liu et al., 2019).

## 4 External factors influencing NeuP

### 4.1 Cytokines and neurotrophins from surrounding cells

After peripheral nerve injury, the surrounding cells (i.e., glial, immune, and tissue cells) undergo changes to promote neuronal regeneration. Notably, Schwann cells organize themselves in Büngner bands to serve as guideposts for sprouting axons (Ribeiro-Resende et al., 2009). Meanwhile, perineuronal satellite cells and resident macrophages proliferate to support regeneration (Lindborg et al., 2018; Feng et al., 2023; Konnova et al., 2023).

All these cells release cytokines (e.g., gp130, IL-6, TGFβ), neurotrophins (e.g., FGF-2, NT-3, NGF and GDNF) and other mediators. These released factors on one hand dampen pain perception, on the other sensitize cells to fire action potentials, promoting peripheral/central sensitization and chronic NeuP (Figures 2, 3; Krames, 2014). Notably, trophic factors like NGF have peculiar mechanisms of action on DRG neurons, as they regulate development, plasticity, cell death, and survival (Lykissas et al., 2007; Khan and Smith, 2015). However, excessive NGF sensitize nociceptors and cause hyperalgesia and/or allodynia in both human and murine models by eliciting pro-inflammatory responses and by increasing the expression of voltage-gated sodium channels (Barker et al., 2020).

The contribution of glial cells to NeuP is extensively studied. In mice models, Schwann cells promote an inflammatory response by releasing ATP through the Panx1 channels and by recruiting T-cells through the expression of MHC II (Hartlehnert et al., 2017; Wang et al., 2022). In rodents, satellite glial cells also release ATP and potassium, which increase neuronal excitability and promote peripheral sensitization (i.e., hyperalgesia; McGinnis and Ji, 2023).

Macrophages phagocyte the endosomes released by damaged DRG neurons and, in response, secrete pro-inflammatory cytokines and NGF, giving rise to and sustaining mechanical allodynia (Simeoli et al., 2017; Green et al., 2019; Yu et al., 2020). Indeed, the DRG-resident macrophages are critical contributors to both the initiation and maintenance of NeuP in rodents (Yu et al., 2020). Upon peripheral nerve injury, these macrophages assume M1 phenotype to produce pro-inflammatory peptides (e.g., IL6, IL-1β, TNF-α, IGF-1) that exacerbate NeuP by increasing the nociceptors excitability (Zhao et al., 2023). In the late stage of nerve damage, regulatory T cells influence the M1/M2 polarization of the macrophages through the release of cytokines. This promotes a shift toward the M2 macrophage phenotype, which alleviates pain and favors axon outgrowth in rats (Chen et al., 2022). Indeed, the anti-inflammatory M2 macrophages secrete high amount of opioid peptides (such as β-endorphin, Met-enkephalin, and dynorphin A) that reduce allodynia in mice (Labuz et al., 2009; Pannell et al., 2016).

Cells localized in the innervated tissue can also affect NeuP, though the studies are limited in number. Murine and human fibroblasts and keratinocytes release NGF, IL-6 and ATP to alter neuronal activity and promote NeuP (Baumbauer et al., 2015; Shinotsuka and Denk, 2022; Xu et al., 2022). In mice, fibroblasts secrete SMOC2, a component of basement membrane, that is necessary for basal mechanical nociceptive threshold in the DRG. By interacting with P2X7 receptor expressed on satellite glial cells, SMOC2 inhibits the coupled activation of adjacent DRG neurons, which in turn suppresses the nociceptive signaling (Zhang et al., 2022). Peripheral inflammation actually causes SMOC2 downregulation in DRG, which exacerbates mechanical allodynia. Fibroblasts also release Protease Inhibitor (PI)16 that that promotes NeuP development by altering the blood-nerve barrier permeability and the leukocyte infiltration (Singhmar et al., 2020; Garrity et al., 2023). PI6 may be an optimal target for new analgesics as (a) it has a limited distribution and (b) in its absence mice are protected from NeuP development (Singhmar et al., 2020). Even adipocytes can influence pain. In mice with nerve damage, adipocytes release adipokine leptin that not only causes allodynia by activating macrophages, but also promotes Schwann cell metabolic adaptation to favor nerve repair (Maeda et al., 2009; Sundaram et al., 2023).

### 4.2 Effect of ECM and substrate on axonal growth

The extracellular matrix (ECM) provides structural support and maintenance of cellular regulation. In particular, ECM influences differentiation, survival, growth and migration. Neurons, like other cells, have receptors on their plasma membrane to interact with ECM components. These are principally glycoproteins (both collagenous and non-collagenous proteins) and proteoglycans secreted by cells in the vicinity. In the case of human and murine DRGs, the ECM elements are principally produced by fibroblasts and neuronal cells (Vroman et al., 2023).

ECM mechanical properties, such as substrate stiffness, module sensory neuron axonal outgrowth and morphology (Roumazeilles et al., 2018). DRG neurons are mechanosensitive cells and their morphology varies according to the stiffness of the substrate (Rosso et al., 2017). The stiffness is perceived through the activation of Piezo1 channel, which induces a calcium influx that regulates E-cadherin and integrin-β1 functions to modify the neuronal cytoskeleton (Lei et al., 2023). Softer substrates actually favor the neurite branching of DRG neurons (Koch et al., 2012) by contrasting the effect of Sema3a, a guidance cue that induce growth cone collapse. In fact, the expression of Sema3a receptors Nrp1 and Plxna4 is controlled by stiffness: stiffer substrates increase Nrp1 mRNA levels while reducing the amounts of Plxna4 mRNA (Vela-Alcatara et al., 2022).

While in normal conditions, the ECM environment support nerve maintenance, when an injury occurs the ECM shifts toward a pro-regenerative status to favor axonal sprouting. *In vitro* studies highlighted how collagen, fibronectin and laminin can differentially affect the neurite outgrowth of sensory neurons and their remyelination post-injury (Baron-Van Evercooren et al., 1982; Deister et al., 2007; Yu et al., 2023). Interestingly, combining ECM components with neurotrophins promotes sensory axons regeneration and target reinnervation. Indeed, treating rats after sciatic nerve injury with a combination of collagen, laminin matrix and NGF/NT3 could regenerate sensory neurons and improve sensory functional recovery (Santos et al., 2017). Notably, chicken DRG *in vitro* culture manifested differences in growth as a response of either NGF or NT3 treatment, depending on the ECM substrate composition they were cultivated on (Guan et al., 2003).

There are increasing evidences that alterations in ECM molecules/pathways are associated with painful conditions. For example, in CIPN models (specifically Drosophila and murine sensory neurons) nociceptive neurons showed altered branching pattern as a result of integrins overexpression (Shin et al., 2021). In addition, after peripheral nerve injury, some types of collagen (i.e., col4α5, col18α1, col19α1) are found upregulated at the damaged site (Roumazeilles et al., 2018). Interestingly, even samples of people suffering from NeuP presented dysregulation of these ECM-genes (Vroman et al., 2023).

## 5 Therapeutic approaches for NeuP targeting the PNS

The pursuit of new drugs for NeuP poses significant challenges, considering the complexities of pain mechanisms and the limitations of existing treatments. Pain-suppressing agents like gabapentin and pregabalin, that block ion channels, can have adverse effects such as somnolence and nausea (Attal, 2019). Opioids, while effective, are associated with addiction and mortality concerns (Neuman et al., 2019; Campbell et al., 2020). The economic burden of pain management is substantial, amounting to $18.3 billion for prescription analgesics and $2.6 billion for non-prescription analgesics in the US only (Turk and Patel, 2022). Finding safer and more effective alternatives is a priority for the pharmaceutical industry.

New approaches to block NeuP at the injury sites are being tested, taking into consideration the recent advances in the field. In a few trials to impair signaling transmission, botulinum toxin A was injected and the patients reported analgesic effects (Attal et al., 2016). Local DRG stimulation with electrodes has also been tested to block pain signaling, but at the moment there is not enough evidence to support its efficacy as a treatment (Knotkova et al., 2021). The VX-548 drug, a NaV1.8 channel inhibitor acting on the PNS, is showing promising results in the clinical trials (Jones et al., 2023). Gene therapies and cellular reprogramming approaches have been tested as a way to achieve analgesia and to promote nerve regeneration, with mixed results (Carvalho et al., 2019; Park et al., 2019). In mice, the targeted ubiquitination of a calcium channel, achieved by viral delivery of a genetically modified protein in DRG neurons, could actually abate hyperalgesia in response to nerve injury (Sun et al., 2022).

Even modulation of the growth factors signaling has been explored as a possible therapeutic method (Li et al., 2020). Tanezumab, an inhibitor of NGF, could reduce lower back pain and diabetic neuropathy, however it was not effective in treating postherpetic neuropathy (Patel et al., 2018). In preliminary studies, neurotrophic factors combined with ECM components were able to enhance sensory axons regeneration and promote appropriate target reinnervation in rat (Santos et al., 2017). Decellularized ECM-structures without growth factors are being tested in rodents to ameliorate the recovery post nerve injury. The results vary, as some boosted neovascularization but not axonal regrowth, while others improved electrophysiologic response and axon counts (Ren et al., 2018; Meder et al., 2021).

More technological approaches are being experimented to alleviate NeuP, such as 3D-bioprinted implantable devices to promote nerve guidance (Sanchez Rezza et al., 2022). Combined expertise of biomechanics, biology and bioengineering will be crucial to develop new implants and achieve complete functional recovery.

## 6 Conclusion

Millions of people worldwide suffer from neuropathic pain (NeuP), which has a huge cost on the healthcare systems and reduces the quality of life and the lifespan of the individuals. This problem is also underestimated as there are not many studies that take into consideration the differences in pain perception between man and women and the effect of aging.

Even though pain perception involves both central and peripheral nervous system, in this review we focalized only on the latter. In particular, we explored what happens after damage of the axons innervating tissue and viscera, while only briefly mentioning the signaling in the spinal cord region.

Peripheral sensory neurons, have a crucial role in pain perception as the initiators of the injury signal. These cells are heavily influenced by extrinsic factors released by neighboring cells (i.e., immune, glial, tissue cells) and by the activation of intrinsic elements (e.g., signaling cascades, axonal-soma communication). The cross-talk between intrinsic and extrinsic factors dictate the outcome of the regenerative program after nerve injury. Any alteration can lead to failure of organ innervation and functional recovery, giving rise to neuropathic pain. The ability to control axonal growth and directionality, while limiting the firing potential (that causes the release of painful stimuli), could be highly beneficial for patients suffering from chronic pain. Discovering new drugs that specifically target the peripheral nervous system should be a priority, as this approach may help manage pain more effectively without affecting central nervous system functions. Such targeted therapies could provide relief by modulating the peripheral mechanisms of pain without the side effects associated with broader systemic treatments.

## Author contributions

LT: Data curation, Investigation, Methodology, Writing – original draft, Writing – review & editing. SD: Data curation, Investigation, Writing – review & editing. LM: Conceptualization, Data curation, Funding acquisition, Investigation, Methodology, Supervision, Writing – original draft, Writing – review & editing. AV: Writing – original draft.

## Footnotes

^1^ http://dolorisk.eu/

## Supplementary material

SUPPLEMENTARY TABLE 1 Information on the NeuP population studies. Here we report the studies per country used to create Figure 1. We were able to find only nineteen studies in the literature that investigated the prevalence of NeuP in the general population. These studies greatly varied in the number of cases examined, the use of controls, and the way the NeuP was evaluated. Moreover, different types of questionnaire to define NeuP were used, such as LANSS, DN4, PainDETECT. While all the questionnaires are extensively used as diagnostic tools, they don’t measure the same parameters. All these factors may partially explain the great variation in NeuP prevalence between countries.
